# Achieving nanosecond time resolution with a two-dimensional X-ray detector

**DOI:** 10.1107/S1600577525006599

**Published:** 2025-08-18

**Authors:** Yuriy Chushkin, Jonathan Correa, Alexandr Ignatenko, David Pennicard, Sabine Lange, Sergei Fridman, Sebastian Karl, Björn Senfftleben, Felix Lehmkühler, Fabian Westermeier, Heinz Graafsma, Marco Cammarata

**Affiliations:** a ESRF – The European Synchrotron, 71 avenue des Martyrs, 38000Grenoble, France; b Deutsches Elektronen-Synchrotron DESY Notkestr. 85 22607Hamburg Germany; c Center for Free-Electron Laser Science CFEL Deutsches Elektronen-Synchrotron DESY Notkestr. 85 22607Hamburg Germany; d Friedrich Schiller University Jena, 07743Jena, Germany; e University of Erlangen-Nuremberg, Schlossplatz 4, 91054Erlangen, Germany; f European XFEL GmbH Holzkoppel 4 22869Schenefeld Germany; g The Hamburg Centre for Ultrafast Imaging, Luruper Chaussee 149, 22761Hamburg, Germany; University of Essex, United Kingdom

**Keywords:** X-ray detector, XPCS, Timepix4, photon science

## Abstract

The fastest pixel array X-ray detectors can record images with nanosecond resolution. This is accomplished by storing only a few images in in-pixel memory cells. In this study, we demonstrate nanosecond resolution over a large number of images by operating a prototype detector in an event driven mode. The performance of this mode is tested by measuring the Brownian dynamics of colloidal nanoparticles. We can achieve sub-100 ns time resolution and overcome the pixel dead time by applying a cross-correlation analysis of the neighboring pixels. The approach used in this work can be extended to study time-resolved fast processes with diffraction, scattering or imaging techniques.

## Abstract 2

A proof-of-principle of a nanosecond time-resolved experiment is demonstrated using a TEMPUS detector. The concept exploits an event driven mode of the detector and cross-correlation analysis to overcome pixel dead time, and offers new opportunities for time-resolved studies.

## 1. Introduction

**Figure 1:**
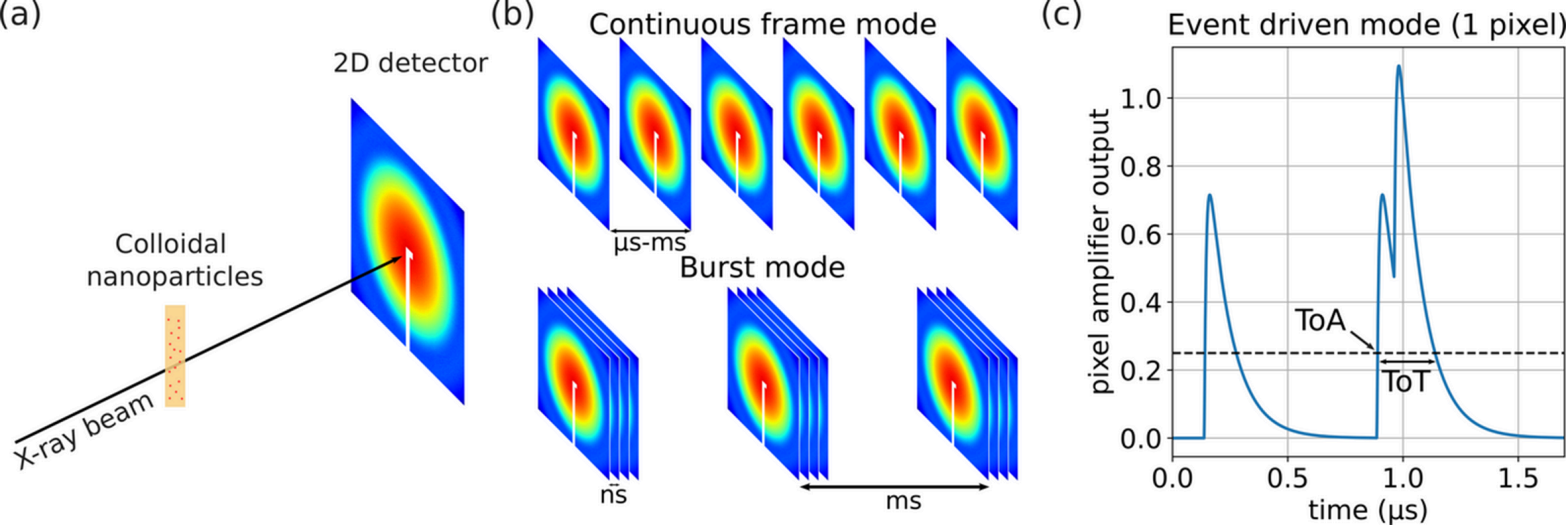
Detector operation modes. (*a*) A sketch of the small angle scattering geometry used in this study. (*b*) Typically temporal series of 2D scattering patterns can be collected in two different acquisition modes (continuous and burst). (*c*) In event driven mode, time of arrival (ToA), time over threshold (ToT) and pixel index are sent in a data packet for each detected photons. In this sketch (1 pixel detector), two events are shown including one and two photons event. Two-dimensional frames can be reconstructed by software counting the photon events within a given time interval.

Bright X-ray sources such as synchrotron radiation sources and free-electron lasers (XFELs) have enabled experiments that can probe equilibrium as well as out-of-equilibrium phenomena occurring on the sub-millisecond time scales. Capturing ns and µs processes happening at nano and atomic length scales can shed light on the functioning of proteins, atomic diffusion in solids and liquids, crystallization in amorphous materials, kinetics and transport phenomena in batteries, *etc*. In most time-resolved experiments, large two-dimensional pixel array detectors are used for fast data acquisition (Hatsui & Graafsma, 2015 ▸; Förster *et al.*, 2019 ▸) [Fig. 1 ▸(*a*)]. Modern state-of-the-art detectors can record continuously a few thousand frames per second [Fig. 1 ▸(*b*)]. Reaching micro- and nano-second times poses a challenge to detector developers (Gruner *et al.*, 2023 ▸). The large number of pixels (> hundreds of thousands) combined with high frame rate results in a large data rate often exceeding the high-speed data readout links and/or writing speed of high-performance storage systems.^1^

Although raw data sparsification has been implemented (Zhang *et al.*, 2021 ▸) to reduce the data rate that needs to be written to disk, the limited bandwidth of the detector head still constrains the frame rate. To overcome this bottleneck, several strategies have been conceived including so-called burst mode (Allahgholi *et al.*, 2019 ▸) [Fig. 1 ▸(*b*)]. In burst mode a detector registers a few [Icarus (Hart *et al.*, 2019 ▸)] to a few tens [Keck-PAD (Philipp *et al.*, 2016 ▸), UFXC32k (Zhang *et al.*, 2018 ▸), Jungfrau (Sikorski *et al.*, 2023 ▸)] or hundreds [AGIPD (Jo *et al.*, 2021 ▸)] of frames in in-pixel memory cells. A significantly slower readout then follows. Although burst mode has been used to achieve nanosecond time resolution, its low duty cycle (often below a few percent) or maximum number of frames compromises its general applicability.

A new alternative approach, tested here, exploits the event driven operation mode implemented in the Timepix chip family (Llopart *et al.*, 2007 ▸; Yousef *et al.*, 2017 ▸; Llopart *et al.*, 2022 ▸; Correa *et al.*, 2024 ▸). In this mode, each single photon detection generates a data packet. Each packet contains information that characterizes the event including time of arrival (ToA) with respect to a trigger signal, time over threshold (ToT), and pixel index [Fig. 1 ▸(*c*)]. This approach optimizes the use of the high-speed network link by transferring only the content of pixels that have detected photons. The latest version of the Timepix family (Timepix4) has a sub-nanosecond timestamp capability and a bandwidth that supports 1.28 billion hits per second (for each chip consisting of 448 × 512 square pixels) (Llopart *et al.*, 2022 ▸) which makes this technology outstanding for fast data acquisition. It should be noted that event driven mode is only possible thanks to the development of Application-Specific Integrated Circuits (ASICs) that power most modern hybrid pixel detectors (Heijne, 2001 ▸). ASICs allow amplifying and reading each pixel of a semiconductor sensor material with low noise and high quantum efficiency.

In this contribution, we demonstrate how the event driven mode can revolutionize time-resolved experiments at a fourth generation synchrotron radiation source (Raimondi *et al.*, 2023 ▸) by applying it to X-ray photon correlation spectroscopy (XPCS). XPCS is the dynamic light scattering (DLS) counterpart in the X-ray photon energy domain. Like DLS, XPCS experiments are intrinsically time-resolved. They consist of recording fast time series of speckle patterns. Speckles are constructive and destructive wave interference that decorate the average far-field scattering pattern when fairly coherent light illuminates a disordered material. Given a short enough exposure, a speckle pattern encodes the instantaneous positions of scatterers. Changes in the positions of the scatterers results in fluctuations in the speckle intensity. Sample dynamics are then quantified by calculating a temporal intensity autocorrelation function (ACF) from a series of speckle patterns. Owing to the small wavelength, XPCS can probe dynamics from micrometre (Möller *et al.*, 2016 ▸; Zinn *et al.*, 2018 ▸) down to angstrom length scales (Leitner *et al.*, 2009 ▸; Ruta *et al.*, 2012 ▸).

The progress of XPCS capabilities relies on bright X-ray sources, such as modern synchrotron radiation sources and XFELs, since they provide X-ray beams with a high degree of coherence (Raimondi *et al.*, 2023 ▸). Another key element has been the development of detectors with high spatial and temporal resolution (Hatsui & Graafsma, 2015 ▸; Förster *et al.*, 2019 ▸). The application of 2D detectors for XPCS has become pivotal as it has numerous advantages. It improves the signal-to-noise ratio (Dierker *et al.*, 1995 ▸; Lumma *et al.*, 2000 ▸; Falus *et al.*, 2004 ▸), allows measuring weakly scattering samples (Ruta *et al.*, 2012 ▸; Vodnala *et al.*, 2018 ▸; Chushkin *et al.*, 2022 ▸), and by enhancing the detection efficiency it reduces the required dose on the sample. Two-dimensional detectors have become indispensable for modern XPCS studies in the hundreds of seconds down to tens of microsecond dynamics, but until now accessing faster times has been difficult due to the limited frame rate of detectors.

Nowadays, the state-of-the-art detectors can operate at microsecond (Zinn *et al.*, 2018 ▸; Cipiccia *et al.*, 2024 ▸) down to sub-microsecond time resolution (Zhang *et al.*, 2018 ▸; Jo *et al.*, 2021 ▸). The fastest XPCS measurements at 195 ns were reported with the AGIPD detector exploiting the pulse time structure of a storage ring together with the 352 memory cells available on each pixel (Jo *et al.*, 2021 ▸). Thus the maximum period that could be probed was about 700 µs.

Here we demonstrate that the TEMPUS (Timepix4-based Edgeless Multi-Purpose Sensor) detector (Correa *et al.*, 2024 ▸) can achieve 20 ns time resolution in XPCS experiments, in principle with an unlimited time window (20 s in this study equating to over nine orders of magnitude). Such time resolution, smaller than the dead time of a pixel, can be attained by cross-correlating events of neighboring pixels. The ns resolution can also be obtained in diffraction, scattering and imaging applications, opening new opportunities for time-resolved studies.

## 2. Methods

### 2.1. X-ray measurements

X-ray measurements were conducted at the ESRF-EBS beamline ID10 using 8 keV (λ = 1.55 Å) radiation selected from the undulator radiation by a Si(111) monochromator. High harmonics were rejected by a double reflection from Si mirrors tuned at a 3.2 mdeg incidence angle. The coherent fraction of the beam was cut out by roller blade slits open to *S*_beam_ = 10 µm and placed 0.5 m upstream of the sample. The coherent beam intensity at the sample position (*I*_0_) was 2 × 10^10^ photons s^−1^. The machine was operating in the uniform filling mode consisting of 992 bunches (100 ps duration) spaced by 2.82 ns at 200 mA current. We used 45 nm Au colloidal nanoparticles coated with ∼8 nm PEG ligands with a hydrophobic 11-mercaptoundecanoic-acid-based spacer (Schulz *et al.*, 2013 ▸; Schulz *et al.*, 2016 ▸). The colloids were suspended in water at 0.05% volume fraction and sealed in 1.5 mm quartz capillary. The small angle scattering was measured by a CdTe Eiger2 4M detector placed at *d* = 7.05 m downstream from the sample. The dynamics of the system were measured by the TEMPUS detector placed at *d* = 7.05 m operating in event driven mode (Correa *et al.*, 2024 ▸).

### 2.2. Detector operation

The TEMPUS detector consists of 448 × 512 pixels each of 55 µm × 55 µm in size. It can be operated in a continuous frame (at 40 kHz) or event driven mode with photon time binning accuracy of 195 ps (Llopart *et al.*, 2022 ▸). During the experiment, only the event driven mode was used. The active sensor is 300 µm thick p-on-n silicon material. The detector was operating at 200 V bias voltage and the threshold was set at 6715 ADU. The total exposure time to collect the data was 20 s for each setting. We tested the detector with different Ikrum values of 5.49, 11.76, 23.53, 47.06 and 94.12 nA. Ikrum is the Krummenacher current in the preamplifier circuit that controls the leakage current and the pulse decay time (Krummenacher, 1991 ▸; Hamann *et al.*, 2015 ▸). The time stamp of simultaneous pulses depends on the amplitude of a signal when a constant threshold is applied. This distortion is called the time-walk and was corrected according to Correa *et al.* (2024 ▸). In this prototype detector only one 1/4 speed data link of 1.28 Gb s^−1^ was active and we were constrained to use 486 pixels from a total of 448 × 512 = 229376 to avoid saturation (see Fig. 1 of the supporting information).

### 2.3. Data processing

The detector recorded the ToA, ToT and corresponding pixel index over 20 s of exposure. This information was split into 2000 × 0.01 s datasets for convenience and then converted to an events list indicating ToA, number of photons, and pixel index based on 1 or 10 ns time bins. Because the number of two- and three-photon hits is less than 1% of single photon hits, we counted them as one photon hit. To obtain the intensity correlation functions *g*^(2)^(*q*, τ) we used an event correlator (Chushkin *et al.*, 2012 ▸). We calculated the temporal intensity autocorrelation function (ACF) according to 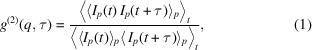
where *I*_*p*_(*t*) is the intensity in pixel *p* at time *t*, and *I*_*p*_(*t* + τ) is the intensity in pixel *p* at lag time τ. 〈…〉_*p*_ denotes averaging over all pixels belonging to the same scattering vector *q*, 〈…〉_*t*_ is the time averaging. In addition we calculated the temporal intensity cross-correlation function (CCF),
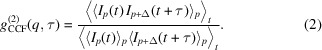
Here *I*_*p*+Δ_(*t* + τ) is the intensity of the neighboring pixel that belongs to the same iso-*q* range of interest. The results presented in this work were obtained by using the nearest neighbor pixel in the horizontal direction but one can also use the nearest neighbor pixel in the vertical direction. Because the measurement of ToA is pixel independent, the CCF can access time intervals shorter than a pixel dead time (see Fig. 2 of the supporting information). Thus, the CCF does not suffer from a pixel dead time in contrast to ACF. All presented correlation functions were converted from linearly spaced to logarithmically spaced in time.

The temporal evolution of the dynamics was monitored using a two-time correlation function (Sutton *et al.*, 2003 ▸),
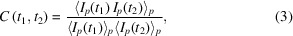
and the time-dependent *g*^(2)^(*q*, τ) can be calculated by averaging *C*(*t*_1_, *t*_2_) over relevant times. Calculation of the correlation functions was performed using an event correlator (Chushkin *et al.*, 2012 ▸) and the width of the iso-*q* range was 0.075 nm^−1^.

## 3. Results

**Figure 2:**
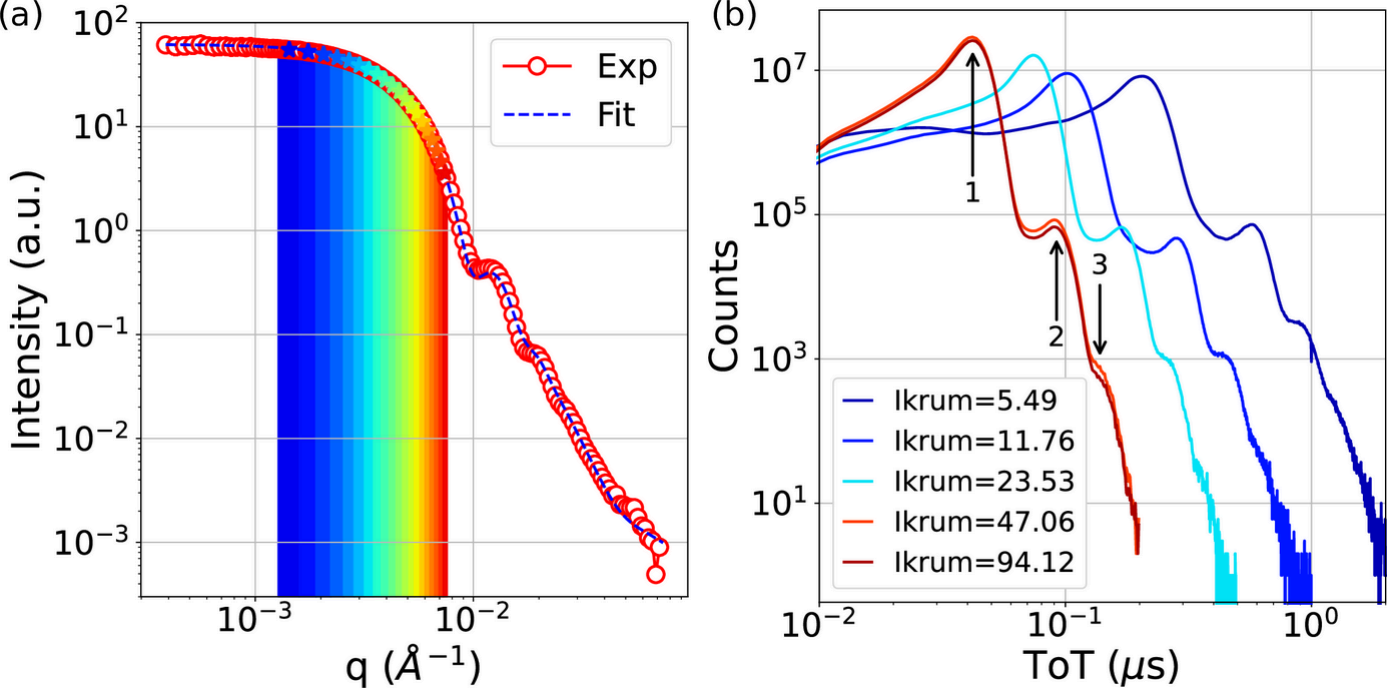
Intensity spatial and temporal distribution. (*a*) SAXS curve of the Au colloidal suspension. Colored stripes indicate the iso-*q* regions where dynamics were measured. (*b*) Distribution of ToT for different Ikrum values in nA. Arrows show the average ToT for 1, 2 and 3 photon events.

The performance of the event driven mode of the TEMPUS prototype detector was tested by measuring the dynamics of dilute colloidal gold nanoparticles suspended in water using XPCS at the ID10 beamline of the ESRF-EBS synchrotron radiation source [Fig. 1 ▸(*a*)]. The nanoparticles undergo Brownian motion resulting in ACFs that can be described by an exponential decay,
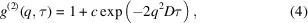
where *c* is the speckle contrast parameter, *q* is the scattering vector, *D* is the diffusion constant that depends on the particle radius *R*, solvent viscosity η and the Boltzmann factor *k*_B_*T*, *D* = *k*_B_*T*/6πη*R*. The relaxation rate is given by Γ = *q*^2^*D*. The small angle X-ray scattering profile of the nanoparticles is shown in Fig. 2 ▸(*a*). The experimental data were fitted with a core-shell model of a gold core radius of 44.3 nm having 13.5% of the Gaussian width of size distribution, and an organic shell thickness of 10 nm. This result is in good agreement with scanning electron microscope measurements of the Au core (44.9 nm) and also matches the DLS data.

Data collection with an event driven mode detector [Fig. 1 ▸(*c*)] consists of ‘listening’ to a dedicated high-speed network link for a given time (in this experiment 20 s for each setting). The prototype used had only one of the 16 high-speed links functional and only at 1/4 of its maximum speed (1.28 Gbit s^−1^). To avoid saturating the link, all but ∼486 pixels covering a large *q*-range were electronically masked during the XPCS data collection (see Fig. 1 ▸ of the supporting information).

The raw data stream was analyzed using scripts that decode each event packet into several pieces of information including the pixel index, ToA and ToT [Fig. 1 ▸(*c*)]. ToA represents the time (from a reference trigger signal) after which the amplified signal crosses a fixed threshold. Conversely, ToT represents the time during which the signal has remained above the very same threshold. The ToT is approximately proportional to the photon energy {or to the number of photons when their time spacing is smaller than the ToT of a single photon [Fig. 1 ▸(*c*)]}.

A histogram of the ToT of events obtained during 20 s data collection is shown in Fig. 2 ▸(*b*) as a function of the Krummenacher feedback current (Krummenacher, 1991 ▸) (Ikrum). The Ikrum sets the speed of the analog amplifier of all pixels. Above 47.06 nA, the speed saturates resulting in a ‘pulse duration’ of ∼40 ns. The histograms contain some events (less than 1%) for which two or more photons are recorded in a single event. Smaller Ikrum results in better photon energy resolution but less time discrimination. It also causes an afterpulsing effect (Molteni & Ferri, 2016 ▸) in ACFs at low Ikrum values as shown in Fig. 5 of the supporting information. Conversely, high values of Ikrum are better suited for high flux applications. It should also be noted that once a pixel has recorded an event it will be unavailable for about 215 ns. This corresponds to the ∼40 ns of the pulse duration with the settings we used, plus 175 ns during which the event is processed and the data packet prepared. This number constitutes the ultimate limit for the ACF on this system.

**Figure 3:**
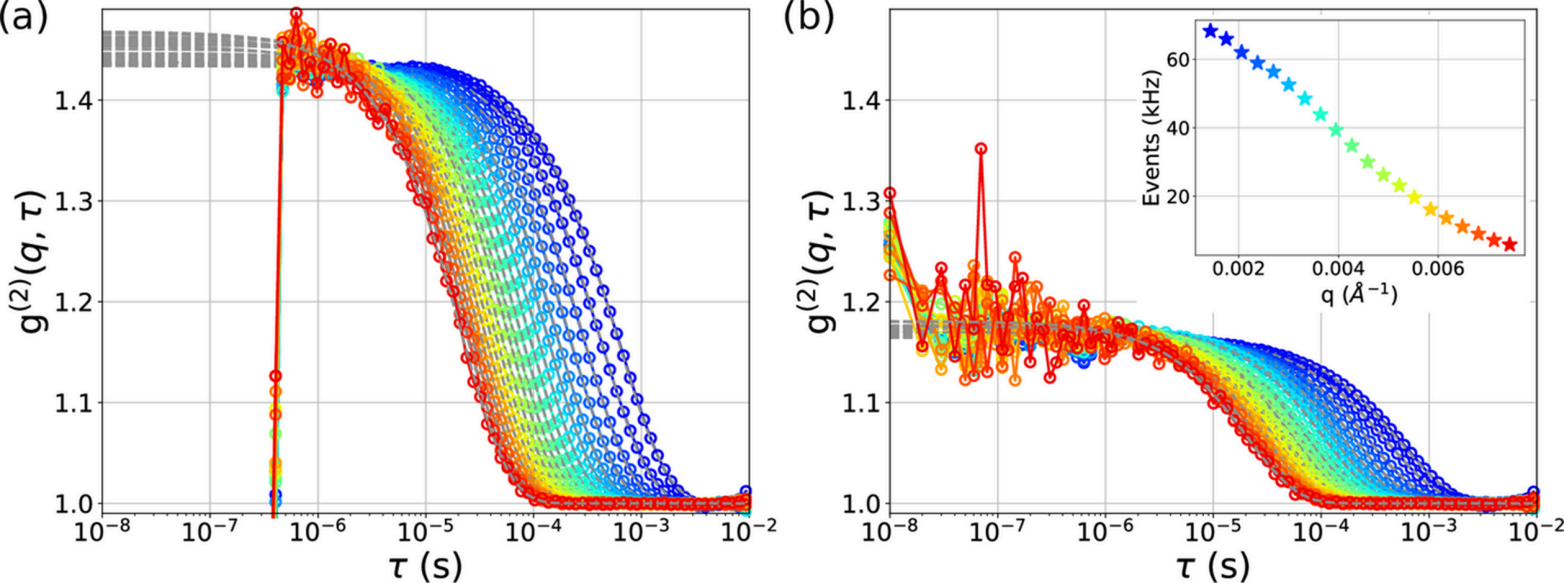
Measured intensity correlation functions. (*a*) Temporal intensity autocorrelation functions (ACF) together with the fits (gray dashed lines). (*b*) Temporal intensity cross-correlation functions (CCF) with the fits (gray dashed lines). The inset of panel (*b*) shows the average count rate for each of the two regions of interest.

More interestingly for XPCS, the collected information on ToA can be used to extract the intensity ACFs using an event correlator (Chushkin *et al.*, 2012 ▸) as discussed in the *Methods* section[Sec sec2]. The ACFs for all 20 *q* values together with the fit to a single exponential decay [equation (4)] are shown in Fig. 3 ▸(*a*). The useful signal starts from ∼450 ns; at the shorter lag time, the values of the ACF drop to zero. This is a direct consequence of dead time at the pixel level. This proves that the event driven mode can be used for continuous (as opposed to burst) data collection at an ‘effective rate’ of 2 MHz. To push the time resolution even further, the pixel dead time limitation needs to be circumvented. This can be done by calculating the intensity cross-correlation function (CCF) (see *Methods*[Sec sec2]) shown in Fig. 3 ▸(*b*). The CCF is often used in dynamic light scattering to suppress multiple scattering (Phillies, 1981 ▸; Meyer *et al.*, 1997 ▸; Zakharov *et al.*, 2006 ▸) or eliminate dead time (Arecchi *et al.*, 1971 ▸; Molteni & Ferri, 2016 ▸). The CCF and ACF functions contain the same information when the following conditions are met. First, the speckle size *S*_speckle_ = λ*d*/*S*_beam_ must be larger than the detector pixel size. From the experimental parameters we estimated the speckle size *S*_speckle_ = 109 µm to be twice the pixel size 55 µm. Thus two neighboring pixels measure independently temporal intensity fluctuations of the same speckle (see Fig. 2 of the supporting information). Second, the average scattering intensity in the nearest neighbor pixels used in the calculation should be similar. Both requirements are fulfilled in our experiment. The CCF has a lower contrast (*c* ≃ 0.14) than ACF (*c* ≃ 0.45); this is expected when the pixel and speckle sizes are comparable.

Intensity auto- and cross-correlation functions were calculated (see *Methods*[Sec sec2]) for 20 iso-*q* values [see colored stripes in Fig. 2 ▸(*a*)]. The Ikrum also affects the short time behavior of the intensity ACFs as shown in Fig. 3 of the supporting information. The Ikrum values 47.06 and 94.12 nA perform equally and we present the results using Ikrum = 47.06 nA.

**Figure 4:**
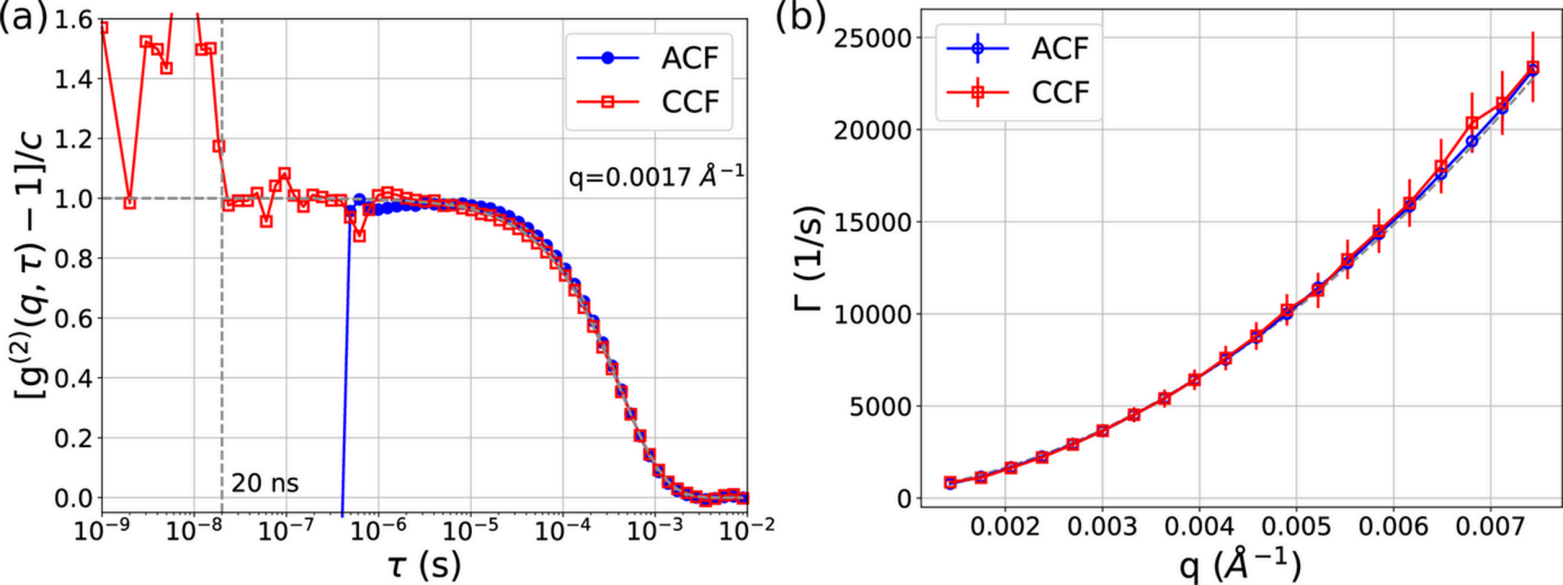
Time performance of the TEMPUS. (*a*) Normalized ACF versus CCF for *q* = 0.0017 Å^−1^ calculated using 1 ns time binning. (*b*) Dispersion curves Γ(*q*) of ACF and CCF with a fit Γ = *Dq*^2^.

A comparison of ACF with CCF is shown in Fig. 4 ▸(*a*). The functions overlap at long times (>100 µs) but there is a slight difference at short times (1–100 µs) where the ACF slightly deviates from the single exponential decay. This is related to the distortion present at high count rates^2^ (Schätzel *et al.*, 1989 ▸). At a full intensity beam condition the detector was operating at the limit of the data transfer speed. Pixels with high count rates may have been losing few data packets causing the observed distortions. The count-rate dependence of the ACF is shown in Fig. 4 of the supporting information where the slight distortion at the higher count rate (∼66 kHz) is visible. The CCFs do not show such a count-rate-dependent effect. Moreover, the use of the CCF allows us to overcome the pixel dead time and extend the correlation function to a shorter lag time. The cross-correlation functions show some artifacts; in particular a small (<15%) artifact around 600 ns and a sudden increase below 20 ns are present in the curves. The origin of these artifacts will need to be investigated further. The 600 ns artifact might be linked to the averaging of pixels belonging to the same ‘super-pixel’ that shares part of the readout circuitry. We expect the feature to be negligible when a large number of pixels are used in calculations.

In XPCS the signal-to-noise ratio (SNR) is given by SNR = 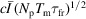 (Lumma *et al.*, 2000 ▸; Falus *et al.*, 2006 ▸), where 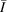 is the average count rate in pixels, *N*_p_ is the number of pixels belonging to a single *q* region, *T*_m_ is the measurement time and τ_fr_ is the exposure time per frame (or the time bin for event driven acquisition). It is thus normal to aim at the highest count rate per pixel given its strong influence on the signal-to-noise ratio. Unfortunately, at a high count rate (>5 × 10^4^ counts) distortions influence the ACF, as shown in Fig. 4 of the supporting information, so care should be taken to avoid them. Interestingly, the CCFs do not suffer from the same artifacts thus allowing the use of higher incoming flux. Previous analysis suggests that for an optimal SNR of the ACF the speckle size should match the pixel size (Falus *et al.*, 2006 ▸). Following this logic, to obtain an optimal SNR in CCF the speckle size should be two times the pixel size.

The correlation functions at each *q* region were fitted with an exponential decay to extract the diffusion constant as shown in Fig. 4 ▸(*b*). This plot also shows that the dispersion relations for ACF and CCF mostly overlap confirming again that the CCF analysis can yield equivalent information to the more classical ACF. For our dataset, the two approaches result in *D* = 4.10 (2) × 10^−12^ m^2^ s^−1^ (ACF) and *D* = 4.14 (6) × 10^−12^ m^2^ s^−1^ (CCF). These results also agree with the DLS characterization that found the particle hydrodynamic radius of 55.1 nm, dispersity index of 0.114, and diffusion constant *D* = 3.93 × 10^−12^ m^2^ s^−1^.

**Figure 5:**
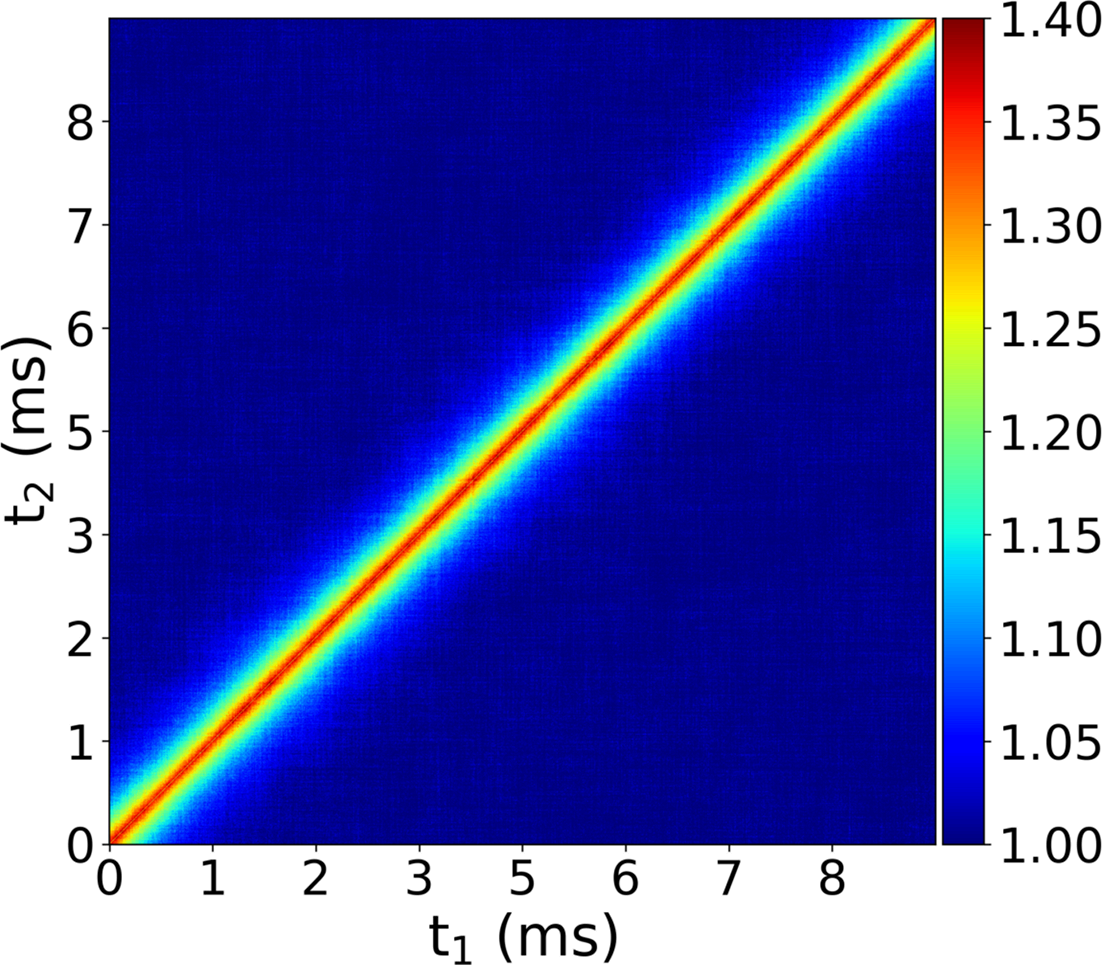
Two-time correlation function for *q* = 0.0017 Å^−1^ calculated using 500 ns time binning.

The photon arrival times can also be used to calculate the two-time correlation function (TTCF) (Sutton *et al.*, 2003 ▸). The TTCF is a time-resolved analog of the intensity correlation function and is used to study non-equilibrium dynamics (Robert *et al.*, 2006 ▸; Ruta *et al.*, 2012 ▸). The TTFC for *q* = 0.0017 Å^−1^ is shown in Fig. 5 ▸. It is a smooth function indicating stationary dynamics characteristic of the Brownian motion of a dilute colloidal suspension. With the high time resolution of TEMPUS data, the TTCF analysis can be extended to study relaxation processes or transient phenomena at nano- and micro-second time scales using a pump and probe approach.

## 4. Discussion

From slow-motion cameras to direct detection detectors for electron and X-ray experiments, novel detectors have revolutionized experimental science in the last decades. Simultaneously, increased source brightness, such as the ESRF-EBS upgrade program, has made high time resolution experiments attainable. One of the techniques that has benefited the most is XPCS thanks to the 30–100 times increase in coherent flux (Raimondi *et al.*, 2023 ▸; Jankowski *et al.*, 2023 ▸) and its favorable SNR scaling with incoming intensity (Lumma *et al.*, 2000 ▸; Falus *et al.*, 2006 ▸). Yet to fully exploit the potential offered by new bright sources appropriately, fast two-dimensional detectors are required (Gruner *et al.*, 2023 ▸).

Our results show how the event driven mode of the hybrid pixel detector unlocks ns time resolution that is not available in the frame mode operation. Despite the TEMPUS detector still being in a prototype phase, we demonstrated that correlation functions down to 20 ns can be measured. The data collected over 20 s exposure were sufficient to obtain good quality correlation functions at ns times (see Fig. 4 ▸). The measured colloidal gold nanoparticles were strongly scattering but we used a low intensity beam and a few hundred pixels to avoid detector data link saturation. When using more pixels and full intensity beam (60 times stronger than used in the experiment), the fast dynamics of less strongly scattering samples can be studied. This opens new possibilities for probing ns dynamics in soft (Sheyfer *et al.*, 2020 ▸) and hard condensed matter (Sandy *et al.*, 2018 ▸), bridging the gap between XPCS, neutron spin echo (Farago, 2006 ▸; Richter, 2006 ▸) and quasielastic X-ray time domain interferometry (Baron *et al.*, 1997 ▸) techniques.

While other existing sub-µs detectors have a limited time window (at best a few hundred times the integration time), the TEMPUS offers a wide period, over nine or more orders of magnitude, covering nanoseconds to seconds times. Such a large time span is required for studies of non-stationary multi-step phenomena or dynamics with multiple relaxations.

When it comes to reaching the fastest times, two factors must be considered: the pulsed nature of the bright X-ray sources and the detector performance. Indeed our results show some artifacts below 20 ns [Fig. 4 ▸(*a*)] even though ASIC can register ToA with 195 ps bin accuracy (Llopart *et al.*, 2022 ▸). This might be caused by the intrinsic uncertainty of the photon detection. X-ray photons are absorbed at different depths in the silicon sensor resulting in different drift times of the charge carrier to the contact. From previous measurements (Correa *et al.*, 2024 ▸) we estimate a time resolution of ∼10 ns which is comparable with our results. The use of high-*Z* sensor materials such as CdTe and GaAs could significantly reduce the aforementioned uncertainty in the absorption depth. This would lead to improved time resolutions. Moreover, the drift velocity of some of these sensors is superior to that of hole-collecting silicon, which is a secondary advantage. Another option to reduce the uncertainty in the absorption depth would be the use of thinner silicon sensors. However, this would result in a lower detection efficiency. There exists a faster alternative to the TEMPUS detector, for example an array of photodiodes (Johnson *et al.*, 2009 ▸; Thil *et al.*, 2012 ▸), but it has a small number of pixels and limited application.

Finally, the time resolution can also be constrained by the interval between X-ray pulses. At the ESRF, the uniform filling mode provides pulses with the shortest spacing of 2.83 ns and the European XFEL is 220 ns (Allahgholi *et al.*, 2019 ▸). A specific data acquisition strategy, similar to the pump–probe measurements, can be conceived to push the time resolution beyond pulse repetition frequency.

Another promising application currently being investigated is the use of TEMPUS for XPCS experiments at XFEL facilities. These experiments are typically performed using detectors with memory cells but with much bigger pixels (*e.g.* 200 µm for the AGIPD) thus significantly decreasing the experimental contrast of the correlation functions for most experimental conditions (Lehmkühler *et al.*, 2020 ▸; Dallari *et al.*, 2024 ▸). Moreover, detectors such as AGIPD are not well suited for high repetition rate quasi-CW XFELs that are being developed around the world (for example, LCLS II-HE in the USA or Shine in China). While XFEL experiments are usually performed with integrating pixel detectors that record multiple photons per pixel per pulse, the ToT capabilities of TEMPUS can also help distinguish events with multiple photons as shown in the ToT histograms of Fig. 2 ▸(*b*). Despite the ToT capability, the TEMPUS detector is not able to handle high count rates (MHz). According to the specification of the Timepix4 chip, the detector can record at an average count rate of 10890 Hz per pixel using full bandwidth (Llopart *et al.*, 2022 ▸). As we show in this work, the count rate can be higher for certain amounts of pixels when others are masked or not hit by a photon. The event driven mode is well suited for fast time-resolved measurements of sparse signals – the domain that is not covered by existing commercial detectors.

## 5. Conclusions

Further detector development and tests are needed to explore the full potential of the event driven mode. Here we tested it with XPCS measurements, but we could find a broad application in other X-ray experiments; for example, in time-resolved studies to track a phase evolution in water ice upon rapid (sub-ms) change in pressure (Pépin *et al.*, 2024 ▸) or the protein structural changes that characterize many proteins at work (Cammarata *et al.*, 2008 ▸). Most of the time-resolved studies reported so far require very special beamlines working with pulsed X-rays (*i.e.* measuring one delay at a time). The use of event driven detectors could enable time-resolved measurements (down to 1 to a few tens of ns) in most high brightness source beamlines.

## Supplementary Material

Supporting figures. DOI: 10.1107/S1600577525006599/rv5195sup1.pdf

## Footnotes

1 The required bandwidth can be estimated for a detector running in continuous mode as *N* × *F* × *B* where *N* is the number of pixels, *F* is the frame rate and *B* is the bit depth of the readout system. A 1 megapixel detector running at 1 MHz at 1 bit pixel^−1^ would result in 1 Tbit s^−1^ data rate.

2 We refer to ‘high’ count rates when the average interval between photon arrival is not much longer than the pixel dead time, ∼210 ns at Ikrum = 47.06 nA.
