# Supplementary material for: Achieving nanosecond time resolution with a two-dimensional X-ray detector
Source: J Synchrotron Radiat. 2025 Aug 18;32(Pt 5):1220–7. doi: 10.1107/S1600577525006599 (PMC12416413; doi:10.1107/S1600577525006599)
Supplement: Supplementary file 1 [file s-32-01220-sup1.pdf]

JOURNAL OF SYNCHROTRON RADIATION

**Volume 32 (2025)**

**Supporting information for article:**

# Achieving nanosecond time resolution with a two-dimensional X-ray detector

**Yuriy Chushkin, Jonathan Correa, Alexandr Ignatenko, David Pennicard, Sabine Lange, Sergei Fridman, Sebastian Karl, Björn Senfftleben, Felix Lehmkühler, Fabian Westermeier, Heinz Graafsma and Marco Cammarata**

# Achieving nanosecond time resolution with a two dimensional X-ray detector

## Supplementary Information

For XPCS measurement we use 486 pixels to avoid the data link saturation but still cover a large q-range. Fig.1 shows an intensity distribution on TEMPUS detector. The white area is masked pixels and the red cross indicates the direct beam position (center of reciprocal space).

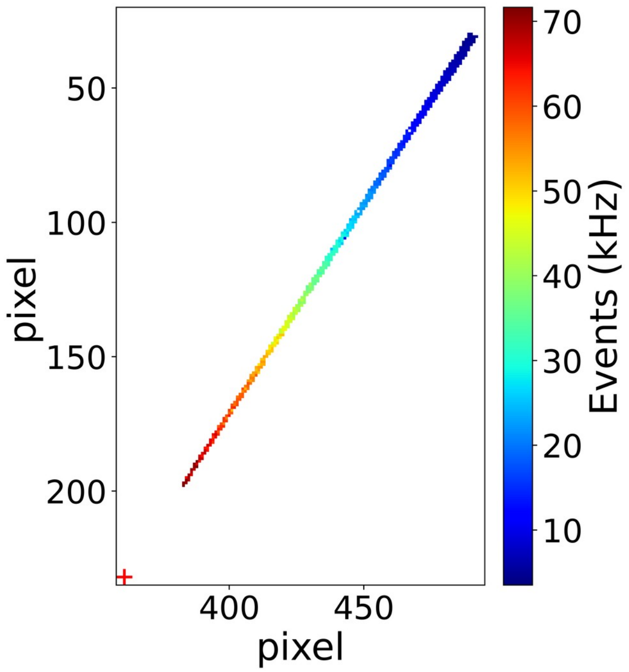


*Fig.1: Intensity distribution in a TEMPUS detector. White area is masked pixels. Number of active pixels is 486.*

Fig.2 displays photon arrival time in a pixel p and its neighbor pixel p+Δ. When each pixel sees independent speckles (top), then the minimum interval time between events is given by a pixel dead time. If two neighboring pixels see the same speckle (bottom), then the minimum interval between events in two pixels is given by the sampling time (less than a pixel dead time).

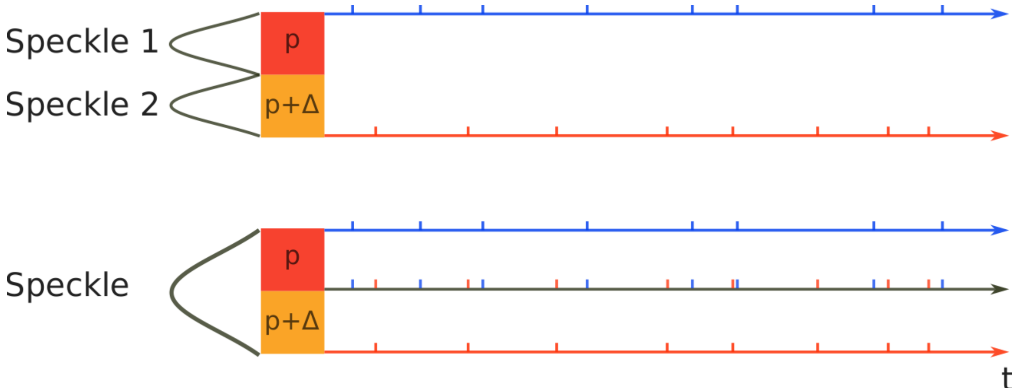


*Fig. 2: Schematics of temporal distribution of photon arrival time in a pixel p and a neighboring pixel p+Δ. Top shows the case for auto correlation, bottom is for cross-correlation between two pixels.*

Fig. 3 shows auto correlation (ACF) and cross-correlation functions (CCF) calculated for different values of Ikrum. The ACFs drop below 1 at short times due to the pixel dead time. The CCFs are extended to few ns times and overcome dead time limitation of the ACFs.

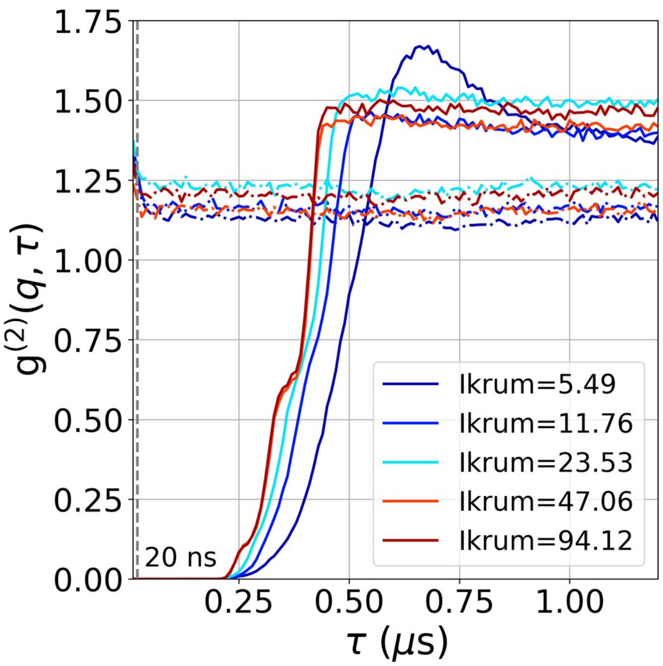


*Fig.3: Intensity correlation function for different Ikrum values in nA. Solid lines are ACFs and dashed lines are CCFs.*

Fig.4 compares the ACFs measured with two different beam intensities. High intensity curve has lower plateau at fast times likely a result of pixels counter pileup.

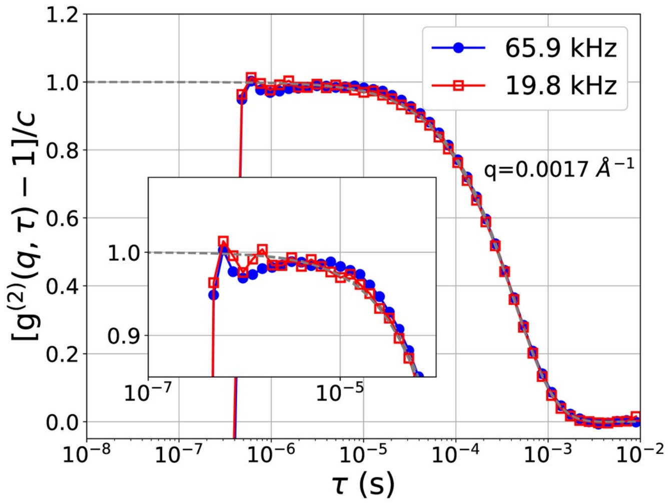


*Fig.4: Comparison of ACF at different fluxes. High flux curve shows the non-linearity distortions related high count rate. Inset is a zoom on short timescale.*

Fig.5 plots ACF at different Ikrum values. A spike around 800 ns at Ikrum=5.49 is a consequence of afterpulsing. This demonstrates that high Ikrum currents are preferred to avoid artifacts in ACFs.

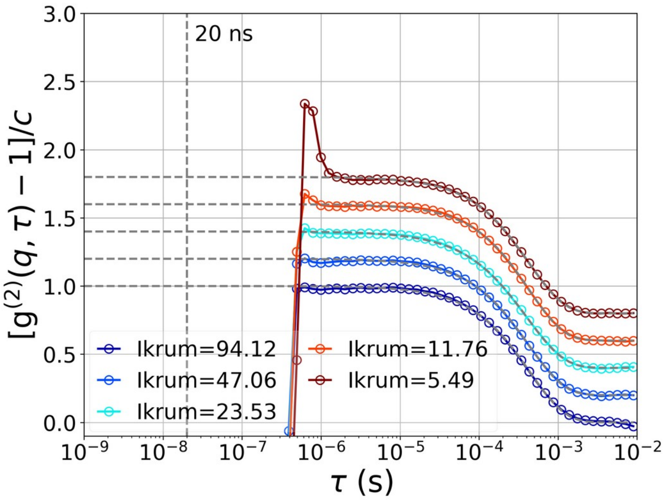


*Fig.5: ACFs at different Ikrum values in nA.*
